# Cervical Spine Range of Motion and Sleep Disturbances in Patients With Atopic Dermatitis: A Cross-Sectional Study

**DOI:** 10.7759/cureus.75449

**Published:** 2024-12-10

**Authors:** Shusaku Hosono, Koji Fujita, Akimoto Nimura, Takuya Ibara, Keiichi Akita

**Affiliations:** 1 Department of Clinical Anatomy, Graduate School of Medical and Dental Sciences, Institute of Science Tokyo, Tokyo, JPN; 2 HealthTech Design Section, Center for Medical Innovation, Institute of Science Tokyo, Tokyo, JPN; 3 Department of Functional Joint Anatomy, Biomedical Engineering Laboratory, Institute of New Industry Incubation, Institute of Science Tokyo, Tokyo, JPN

**Keywords:** allergic responses, atopic dermatitis, cervical spine movement, inflammatory markers, neutrophil-to-lymphocyte ratio, sleep disturbance

## Abstract

Background: Sleep disturbances are common and distressing among patients with atopic dermatitis (AD), often resulting in a cycle of worsening skin conditions. Among various factors affecting sleep in AD, cervical spine movement has been suggested to influence sleep quality; however, these studies mostly relied on subjective measures. Owing to the lack of objective and quantitative analyses of cervical spine movement, its association with sleep disturbances remains poorly understood. This exploratory study aimed to investigate the relationship between quantitatively measured cervical spine range of motion (ROM) and sleep disturbances in patients with AD and to further explore connections with inflammatory and allergy-related markers.

Methods: A cross-sectional study was conducted on 261 patients with AD aged 18-60 years. Sleep disturbances and pruritus were assessed using Numerical Rating Scales (NRS). Cervical spine ROM was quantitatively measured using an automatic measurement system based on flexion-extension X-rays. Hematological parameters, including total serum immunoglobulin E (IgE), thymus and activation-regulated chemokine (TARC), lactate dehydrogenase (LDH), peripheral eosinophil count, and neutrophil-to-lymphocyte ratio (NLR), were analyzed. Group comparisons based on the median cervical ROM and ordinal logistic regression analysis were performed to explore the relationships between these variables.

Results: Patients with higher cervical spine ROM (≥86°) had significantly lower Sleep Disturbance NRS scores and lower levels of inflammatory and allergy-related markers (IgE, TARC, LDH, and NLR) than patients with lower ROM (<86°) (p < 0.05). Ordinal logistic regression analysis revealed that pruritus severity (odds ratio (OR): 3.97, p < 0.001) and age (OR: 1.60, p < 0.001) were positively associated with sleep disturbances, whereas cervical spine ROM was negatively associated with sleep disturbances (OR: 0.72, p = 0.021). TARC levels were positively associated with sleep disturbances (OR: 1.98, p < 0.001).

Conclusions: This study revealed a significant association between cervical spine ROM and sleep disturbances in patients with AD. Higher cervical spine mobility was associated with better sleep quality and lower inflammatory marker levels. These findings suggest the potential of cervical spine interventions in managing sleep disturbances and inflammation in patients with AD. Nonetheless, further research is required to explore the causal relationships and efficacy of targeted interventions.

## Introduction

Sleep management is crucial for the effective treatment and management of atopic dermatitis (AD) [1]. This is because sleep and symptoms such as pruritus and skin manifestations influence each other in a bidirectional manner [2]. Sleep disturbances can worsen AD by triggering systemic inflammation through the immune system [3]. Various factors can contribute to sleep disturbances, including psychological elements such as stress and depression [4], internal organ dysfunction causing visceral sensory stimuli [5], and somatosensory stimuli, such as pruritus from AD and musculoskeletal pain [6,7].

Musculoskeletal pain, particularly in the cervical and shoulder regions, is often linked to sleep disturbances as symptoms worsen [8]. As these complaints worsen, cervical and shoulder mobility tends to decrease [9]. Conversely, improving mobility in these regions can alleviate sleep disturbances [10]. The interplay between cervical mobility and sleep appears to involve autonomic nervous system function [11] and inflammatory responses [12]. While a relationship between cervical spine movement and sleep has been demonstrated by previous studies, their findings are largely based on subjective assessments, such as self-reported pain and subjective mobility [8,9]. Owing to the lack of objective and quantitative analyses of cervical spine movement, its association with sleep disturbances remains poorly understood [8,9].

Traditionally, cervical spine movement is quantitatively assessed by measuring the range of motion (ROM) at each cervical intervertebral level using flexion-extension X-rays [13]. However, this time-consuming process is not suitable for clinical practice. Recently, automatic measurement systems powered by machine learning models have been developed to address this limitation [14].

Establishing an association between sleep disturbances and cervical spine ROM in patients with AD could lead to the development of new intervention strategies for managing sleep disturbances in these patients. This exploratory study aimed to analyze the association between sleep disturbances and cervical spine ROM in patients with AD. We hypothesized that quantitatively measured cervical spine ROM would be associated with sleep disturbances in patients with AD.

## Materials and methods

Study design

We conducted a cross-sectional study using medical records and radiographic data of patients who visited the AD outpatient clinic at Hosono Clinic in Tokyo, Japan, from June 2019 to December 2020. This AD clinic specializes in integrative medicine treatments.

Ethical considerations

This clinical study was approved by the local Ethics Review Committee of Tokyo (approval number: 635023-20181214). All participants provided written informed consent before taking part in the study.

Study criteria

The inclusion criteria were as follows: age 18-60 years, complete questionnaire responses, and no missing blood test data. Thirty-nine patients were excluded owing to age (n = 26), incomplete questionnaire responses (n = 5), and missing data (n = 8). Ultimately, out of 300 patients who provided informed consent, 261 met the study inclusion criteria and were subsequently included in the analysis.

Procedure

Data were collected via questionnaires during the initial outpatient visit to the AD clinic at Hosono Clinic. Age was calculated based on the date of birth at the time of the visit.

Licensed radiological technologists obtained two-dimensional X-ray images of the cervical spine in maximum flexion and extension positions for all study participants during the initial outpatient visit, along with baseline assessments including questionnaires and blood samples. The digital radiographic images were exported in JPEG format, and the total cervical spine ROM was measured using an automatic measurement designed to assess mobility based on these maximum flexion and extension positions [14]. This system recognizes each vertebral body in the lateral views during cervical flexion and extension and automatically measures the ROM between the vertebral bodies.

Hematological parameters such as the total serum immunoglobulin E (IgE) levels, serum thymus and activation-regulated chemokine (TARC) levels, serum lactate dehydrogenase (LDH) levels, peripheral eosinophil count, and neutrophil-to-lymphocyte ratio (NLR) were measured during the initial visit.

Assessments

The Sleep Disturbance Numerical Rating Scale (NRS) is rated on a scale of 0 to 10, with 0 indicating “good sleep” and 10 signifying “no sleep at all” [15]. It is a single-item outcome measure used for assessing the severity of sleep disturbances and directly reflects the subjective sleep quality of patients over the past 24 hours [15].

The Pruritus NRS is a self-reported, single-item outcome measure designed to assess peak pruritus over the past 24 hours [16]. It is rated on a scale of 0 to 10, with 0 representing “no pruritus” and 10 indicating “the worst imaginable pruritus.” This scale provides a sensitive assessment of pruritus severity in patients with AD [16]. The content validity of Sleep Disturbance NRS and Pruritus NRS has been established for AD [15,16].

Higher ROM scores indicate greater flexibility, increased mobility, and reduced stiffness [17].

Total serum IgE levels (normal value: ≤170 IU/mL) serve as an indicator of the long-term response to AD treatment [18]. Serum TARC levels (normal value in adults: 450 pg/mL) correlate with AD severity [19]. Peripheral eosinophil count (normal value: 70-450/μL) can estimate AD severity [20], whereas serum LDH levels (normal value: 105-245 IU/L) can determine the short-term parameter of AD severity [21]. A higher value of these parameters indicates greater AD severity [22].

The NLR was calculated as the ratio of the neutrophil count to the lymphocyte count in peripheral blood. It served as an inflammatory biomarker linking two immune responses, namely, the innate immune response mediated primarily by neutrophils and the adaptive immune response facilitated by lymphocytes [23]. While a precise cut-off value has not yet been established, changes in the NLR over time are believed to indicate immune system abnormalities. An increase in NLR is associated with acute stress, while a decrease suggests a maintained immune balance and correlates with positive prognostic factors across various fields [23].

Sample size calculation

To calculate the sample size for comparing Sleep Disturbance NRS between two groups based on cervical ROM, a minimal clinically important difference of 1 point with a standard deviation of 3 was utilized. Using a two-sided type I error of 0.05 and 80% power, a minimum sample of 284 patients (142 per group) was required.

Statistical analysis

Participant data were expressed as means and standard deviations (SD).

Participants were divided into two groups according to the median cervical spine ROM value of 86° (<86° and ≥86°). The median was chosen as a statistically valid approach for this exploratory study, as it provides an unbiased threshold for equal group sizes [24]. The results of comparisons between the group with good cervical spine mobility (≥86°) and the group with poor cervical spine mobility (<86°) were presented as medians with first and third quartile values and were analyzed using the Mann-Whitney U test.

Subsequently, ordinal logistic regression analysis was conducted as the primary statistical analysis, with Sleep Disturbance NRS as the objective variable and with cervical spine ROM, Pruritus NRS, age, IgE, TARC, and NLR as the explanatory variables. Sleep Disturbance NRS and Pruritus NRS were classified into four levels (namely, NRS 0, 1-3, 4-7, and 8-10) to reflect the severity of symptoms [25]. IgE and TARC were categorized into three levels for statistical analysis. The TARC thresholds of 700 pg/mL and 3000 pg/mL were used to classify patients into moderate (701-3000 pg/mL) and severe (>3000 pg/mL) groups [19]. For IgE, thresholds of 1000 IU/mL and 10000 IU/mL were selected based on reported associations with treatment outcomes [18]. For NLR, six categories were established to reflect the degree of inflammation, ranging from below normal (0-1) to severe (11 and above) [23]. These categorizations were determined based on findings from previous studies.

Age and ROM were categorized by considering the distribution characteristics of the data. Given that the SD of age was 10.1 years, we deemed it appropriate to set the categories at 10-year intervals, establishing five categories from 18 to 58 years old. Considering that the SD of ROM was 17.3°, we divided it into seven categories at approximately 20° intervals, equivalent to approximately 1 SD. Specifically, categories were set to cover the inclusive range from 10° to 130°. These categorizations allowed us to convert each variable into a format suitable for statistical analysis while maintaining clinical significance. The validity of these categorizations will be discussed in detail along with the interpretation of results.

In the ordinal logistic regression analysis, odds ratios (ORs) and 95% confidence intervals (CIs) were calculated to evaluate the impact of each explanatory variable on sleep disturbances. This analysis enabled us to quantitatively assess the influence of each explanatory variable on the severity of sleep disturbances while considering the ordinal nature of the Sleep Disturbance NRS.

All statistical analyses were performed using EZR (Easy R) version 1.67 (Saitama Medical Center, Jichi Medical University, Saitama, Japan). EZR is a statistical software that extends the functions of R and R Commander and is designed to easily perform statistical methods frequently used in biostatistics [26]. The significance level was set at p < 0.05.

## Results

**Figure 1:**
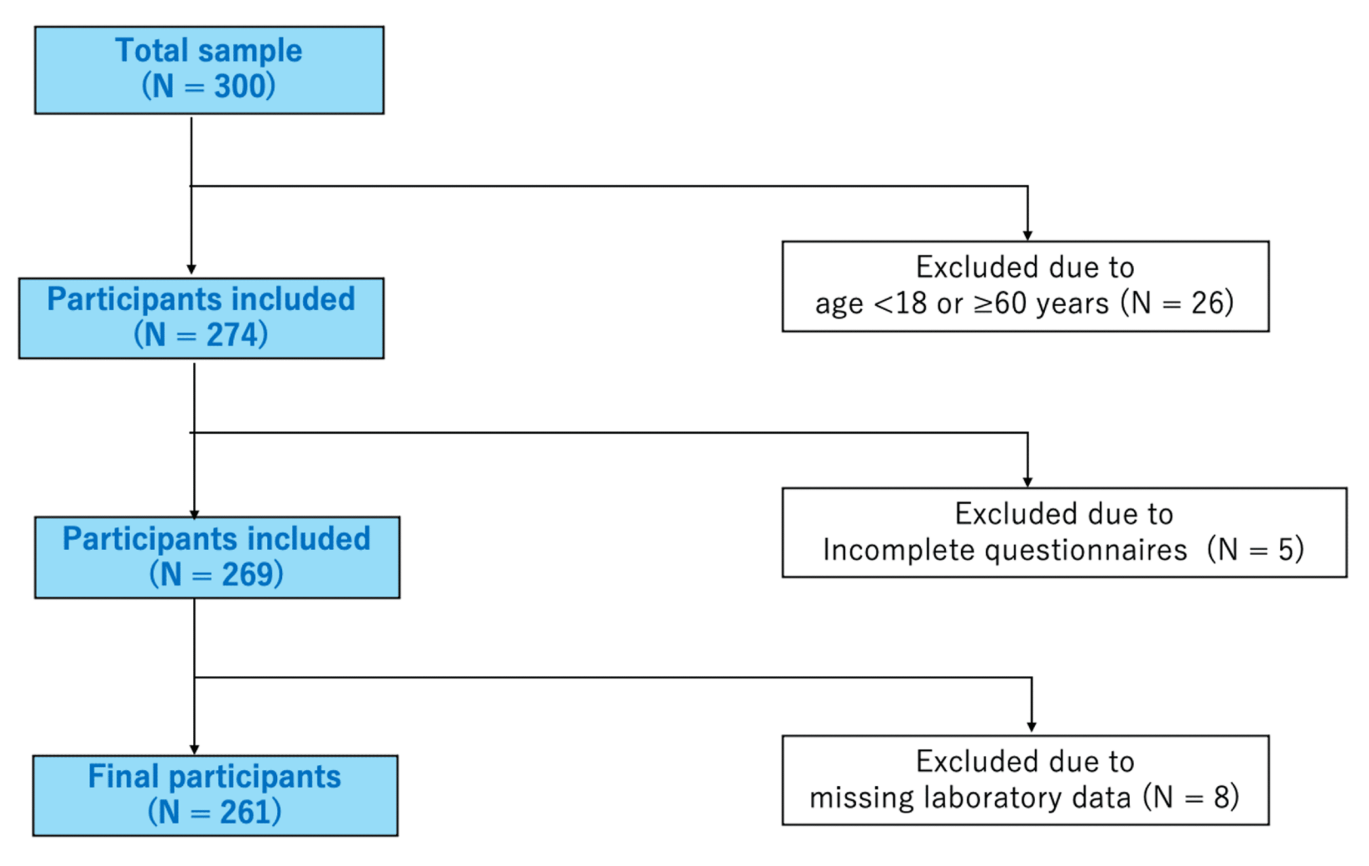
Study flowchart The inclusion criteria used to select participants are displayed. N: number

**Figure 2:**
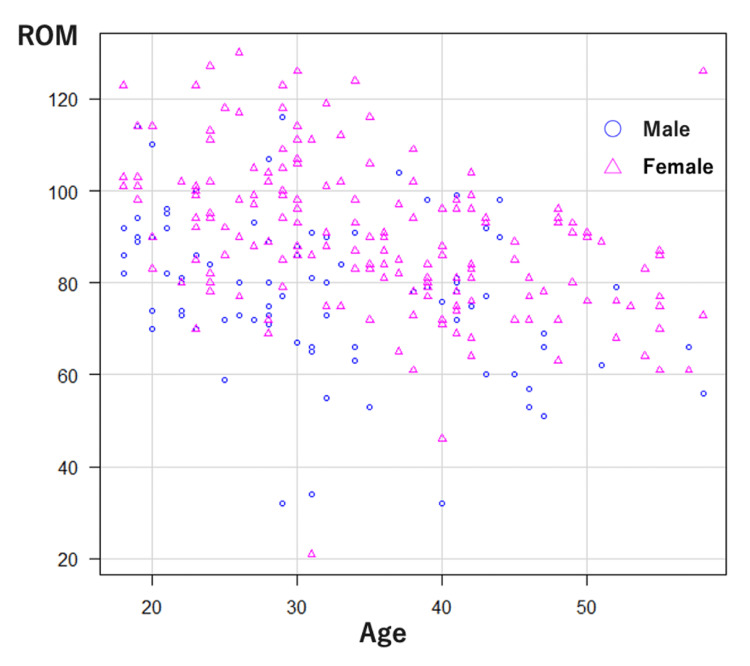
Distribution of cervical ROM by age in male and female participants Circles represent male participants (n = 89), and triangles represent female participants (n = 172). ROM: range of motion

Out of 300 participants, 261 met the age criteria and provided complete responses along with their test results. This group constituted the final dataset for analysis (Figure 1). The mean age of the participants was 34.1 ± 10.1 years (range: 18-58 years). The mean values of Sleep Disturbance NRS, Pruritus NRS, and cervical spine ROM were 4.0 ± 3.0, 6.4 ± 2.5, and 86.0 ± 17.3, respectively (Table 1). The distribution of cervical ROM by age is shown separately for male and female participants (Figure 2).

**Table 1:** Characteristics of study participants Each value is shown as mean ± standard deviation. IgE: immunoglobulin E, LDH: lactate dehydrogenase, N: number, NLR: neutrophil-to-lymphocyte ratio, NRS: Numerical Rating Scale, ROM: range of motion, TARC: thymus and activation-regulated chemokine

Number of participants	261
Male sex, N (%)	89 (34.1)
Age	34.1 ± 10.1
Sleep Disturbance NRS	4.0 ± 3.0
Pruritus NRS	6.4 ± 2.5
Cervical ROM	86.0 ± 17.3
IgE	4621.8 ± 6924.9
TARC	2937.7 ± 5087.6
LDH	260.5 ± 96.8
Eosinophils	742.2 ± 1040.3
NLR	2.62 ± 1.20

**Figure 3:**
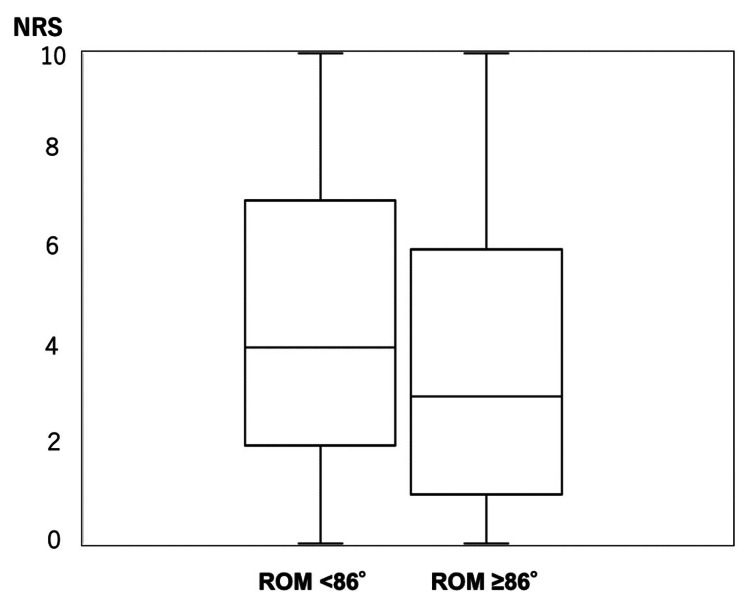
Relationship between Sleep Disturbance NRS and cervical ROM Within each box, horizontal lines denote median values; boxes extend from the 25th to the 75th percentile of each group's distribution of values; vertical lines extend to the maximum and minimum values. NRS: numerical rating scale, ROM: range of motion

The gender distribution showed that the ROM < 86° group included 59 males and 69 females (n = 128), while the ROM ≥ 86° group included 30 males and 103 females (n = 133). The group with higher cervical spine ROM showed significantly lower values in Sleep Disturbance NRS, age, and levels of IgE, TARC, and LDH, as well as NLR, compared to the group with lower ROM. However, no significant differences were observed in pruritus severity or eosinophil counts (Table 2). The distribution of Sleep Disturbance NRS between the two ROM groups is shown (Figure 3).

**Table 2:** Comparison of factors in different cervical ROM Each value is shown as median (first-third quartile). IgE: immunoglobulin E, LDH: lactate dehydrogenase, NLR: neutrophil-to-lymphocyte ratio, NRS: numerical rating scale, ROM: range of motion, TARC: thymus and activation-regulated chemokine *p < 0.05: statistically significant

Factors	ROM < 86°	ROM ≥ 86°	p
Sleep Disturbance NRS	4 (2-7)	3 (1-6)	0.0052*
Pruritus NRS	7 (5-8)	7 (4-8)	0.16
Age	38 (29-44)	30 (24-36)	<0.001*
IgE	2691.5 (425.8-8120.5)	1725.0 (234.0-4209.0)	0.048*
TARC	1160.5 (541.5-3827.3)	795.0 (425.0-2280.0)	0.025*
LDH	245.0 (198.0-315.3)	228.0 (187.0-296.0)	0.038*
Eosinophils	550.6 (333.4-927.0)	429.0 (258.5-832.2)	0.053
NLR	2.46 (1.93-3.37)	2.14(1.67-3.02)	0.015*

Pruritus NRS showed the strongest positive association with sleep disturbances (OR: 3.97, 95% CI: 2.76-5.79, p < 0.001). Age also exhibited a significant positive association with sleep disturbances (OR: 1.60, 95% CI: 1.26-2.05, p < 0.001), while cervical spine ROM showed a significant negative association with sleep disturbances (OR: 0.72, 95% CI: 0.55-0.95, p = 0.021). Additionally, TARC showed a significant positive association with sleep disturbances (OR: 1.98, 95% CI: 1.39-2.84, p < 0.001). No significant associations were found for IgE and NLR (p = 0.73 and p = 0.60, respectively) (Table 3).

**Table 3:** Ordinal logistic regression analysis of factors affecting Sleep Disturbance NRS B: regression coefficient, CI: confidence interval, IgE: immunoglobulin E, OR: odds ratio, NLR: neutrophil-to-lymphocyte ratio, NRS: Numerical Rating Scale, ROM: range of motion, SE: standard error, TARC: thymus and activation-regulated chemokine *p < 0.05: statistically significant

Factor	B (SE)	t value	OR (95% CI)	p
Cervical ROM	-0.32 (0.14)	-2.30	0.72 (0.55-0.95)	0.021*
Pruritus NRS	1.34 (0.19)	7.30	3.97 (2.76-5.79)	<0.001*
Age	0.47 (0.12)	3.83	1.60 (1.26-2.05)	<0.001*
IgE	0.07 (0.19)	0.34	1.07 (0.73-1.57)	0.73
TARC	0.68 (0.18)	3.73	1.98 (1.39-2.84)	<0.001*
NLR	0.08 (0.15)	0.53	1.08 (0.81-1.44)	0.60

## Discussion

In this study involving 261 patients with AD, we first examined the differences between the groups with good and poor cervical spine mobility. Based on the median ROM value, the group with better cervical spine mobility had better sleep quality and lower levels of allergy-related and inflammatory markers. Subsequently, we investigated the relationship between sleep disturbances and various factors through an ordinal logistic regression analysis. Our findings indicated that pruritus intensity was most strongly associated with sleep disturbances (OR: 3.97, p < 0.001); sleep disturbances also exhibited a positive association with both age (OR: 1.60, p < 0.001) and TARC levels (OR: 1.98, p < 0.001). Conversely, a negative association was found between sleep disturbances and cervical spine ROM (OR: 0.72, p = 0.021). IgE levels and NLR did not show significant associations with sleep disturbances (p = 0.73 and p = 0.60, respectively). These results suggest that sleep disturbances in patients with AD are significantly associated with pruritus intensity, age, TARC levels, and cervical spine mobility.

Previous studies indicate an association between cervical complaints and sleep disturbances [9,10]. Furthermore, a relationship between neck pain and sleep quality has been established, with increased neck pain intensity being associated with poorer sleep quality and reduced quality of life [9,10]. Nonetheless, the majority of these studies relied on subjective measures, such as questionnaires and self-reported assessments of pain mobility, and lacked objective quantitative evaluations. In contrast, an automatic measurement system powered by machine learning models was employed in our study to objectively quantify cervical spine ROM. This enabled us to analyze the association between quantitative cervical spine ROM and the severity of sleep disturbances, revealing that reduced cervical spine ROM was significantly associated with sleep disturbances.

Sleep disturbances are associated with systemic inflammation and allergic responses. Disrupted sleep patterns elevate inflammatory cytokine levels, causing systemic inflammation [27]. For instance, patients with poor sleep quality often exhibit higher levels of inflammatory markers, such as C-reactive protein and interleukin-6 [27]. Additionally, the intensity of sleep disturbances is significantly associated with an increased NLR [28]. A positive association between the severity of AD and the intensity of sleep disturbances has also been demonstrated, alongside an association between the intensity of sleep disturbances and the levels of allergy-related markers such as serum IgE [29]. Collectively, these findings suggest a relationship between sleep disturbances, systemic inflammation, and allergic responses [27-29].

The relationship between the quantified cervical spine ROM, sleep disturbances, allergy-related markers, and inflammatory markers was examined in this study. Patients with better cervical spine ROM were associated with milder sleep disturbances and lower levels of allergy-related and inflammatory markers. These findings suggest that the quantified cervical spine ROM is associated with sleep disturbances and key inflammatory and allergy-related markers.

Our findings suggest a complex relationship between cervical spine ROM and autonomic nervous system function. Based on previous studies, restricted cervical spine ROM may cause chronic muscle tension around the cervical region, which may disrupt the autonomic nervous system function [27]. This disruption could affect sleep quality and immune system regulation [28]. While immune system dysregulation might affect cervical ROM through inflammatory responses, the exact mechanisms and directionality of these relationships require further investigation.

As mentioned in the introduction, sleep disturbances are influenced by various factors. While our study focused on quantitative measurement of cervical ROM, psychosocial factors and physical comorbidities may affect both sleep disturbances and cervical mobility through multiple pathways. Depression and sleep disturbances show a bidirectional relationship [4], and neck and shoulder pain is significantly associated with poor sleep quality [9]. Clinical evidence suggests that interventions improving cervical function may enhance sleep quality [30]. Consideration of these confounding factors will be important for future research.

Our study holds important clinical relevance. The results demonstrate a significant association between cervical spine ROM and sleep disturbances in patients with AD, highlighting the critical role of sleep management in patients with AD. Our findings suggest that cervical spine ROM evaluation might be worth considering when assessing sleep disturbances in AD patients. Furthermore, besides conventional topical and pharmacological treatments, complementary interventions aimed at improving cervical spine function through kinematic approaches, such as targeted exercise or physical therapy, may improve quality of life by promoting better sleep, which may result in anti-inflammatory benefits.

Limitations of the study

This study has several limitations. First, as a cross-sectional observational study, it cannot establish causality between the observed variables, only associations. Second, this study was conducted at a single facility specializing in integrative medicine treatments, thereby limiting the generalizability of the findings. Third, we did not collect data on potential confounding factors such as body mass index and use of sleep-affecting medications, which could affect sleep quality. Fourth, the assessment of sleep disturbances was based exclusively on patients' subjective NRS. Lastly, the categorization of continuous variables in our analysis might have resulted in some loss of information.

## Conclusions

Quantitatively measured cervical spine ROM revealed significant associations with sleep disturbances and inflammatory markers in patients with AD. These findings suggest that cervical spine function may be related to sleep quality and inflammatory responses in AD. Future studies should explore causal relationships and potential interventions targeting cervical spine mobility, which could potentially lead to improved sleep, reduced inflammation, and enhanced overall quality of life in patients with AD.
